# Periportal Edema as an Extrarenal Manifestation of Acute Pyelonephritis

**DOI:** 10.3390/diagnostics14060638

**Published:** 2024-03-18

**Authors:** Yu-Yun Chang, Kuei-Hong Kuo

**Affiliations:** 1 Division of Medical Image, Far Eastern Memorial Hospital, New Taipei City 22060, Taiwan; enjoyariel@hotmail.com; 2 School of Medicine, National Yang Ming Chiao Tung University, Taipei 11221, Taiwan

**Keywords:** extrarenal finding, periportal edema, acute pyelonephritis, extrarenal manifestation, right side involvement, clinical outcome

## Abstract

Acute pyelonephritis is a common infection of the upper urinary tract that affects approximately 250,000 adults in the United States. Individuals with acute pyelonephritis require hospitalization and intravenous antimicrobial therapy. Diagnoses of acute pyelonephritis are made on the basis of clinical and laboratory findings. Individuals with complex or severe acute pyelonephritis undergo contrast-enhanced computed tomography (CT) for the diagnosis and assessment of perirenal abnormalities. However, extrarenal manifestations, such as periportal edema and gallbladder wall thickening, may complicate the diagnostic process. We report the case of a 42-year-old woman who presented with fever, dysuria, and flank pain—the hallmarks of urosepsis. CT results confirmed acute pyelonephritis accompanied by periportal edema and elevated levels of hepatic enzymes and C-reactive protein. Despite antibiotic intervention, febrile episodes persisted for 4 days and abated over a fortnight. The patient’s blood and urine cultures yielded negative results, which may be attributed to her prior antimicrobial treatment. Recognition of extrarenal signs in acute pyelonephritis is crucial for obtaining accurate diagnoses and understanding their clinical implications.

**Figure 1:**
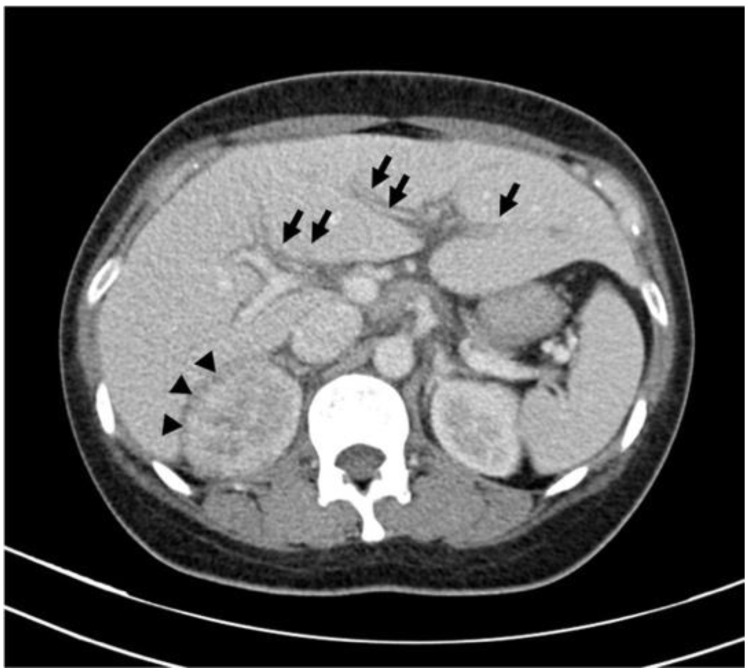
Axial contrast-enhanced computer tomography scan of abdomen reveals compromised corticomedullary differentiation and hypoenhanced, wedge-shaped lesion in right kidney (indicated by arrowheads), which are characteristic of acute pyelonephritis. Additionally, reduced attenuation can be observed in periportal region (indicated by arrows), which is indicative of periportal edema. These imaging findings confirm right-sided acute pyelonephritis accompanied by periportal edema. Gallbladder (not shown) is normal in appearance. Contrast-enhanced computed tomography (CT) has become a useful tool for evaluating perirenal findings, including abscesses, pyonephrosis, xanthogranulomatous pyelonephritis, tuberculosis, and calculi, particularly in the presence of symptoms or signs indicating complication [1]. Extrarenal manifestations, particularly periportal edema and gallbladder wall thickening, can lead to diagnostic challenges. Herein, we report the case of a 42-year-old woman who presented to the emergency room with a 5-day history of fever, dysuria, urinary frequency, myalgia, and right flank soreness. A clinical evaluation suggested urosepsis, as evidenced by the patient’s relative tachycardia (heart rate, approximately 100 bpm), hypotension, tachypnea, and significant pyuria (urine white blood cell count, 5265/HPF; bacteria, 248.9 × 105/mL). Contrast-enhanced CT confirmed the diagnosis of right-side acute pyelonephritis and periportal edema. Elevated levels of alanine aminotransferase (66 U/L) and C-reactive protein (CRP; 22.28 mg/dL) and mild hyponatremia (Na, 130 mmol/L) were noted. Although initial antibiotic therapy was administered (third-generation cephalosporin), the patient’s fever persisted for 4 days after admission to the emergency room. The patient’s liver enzyme levels normalized over 2 weeks. The patient’s 10-day hospital stay concluded with a full recovery. Acute pyelonephritis predominantly presents unilaterally, with a higher incidence of right-sided involvement [2]. The spectrum of renal and extrarenal manifestations in acute pyelonephritis includes renal and perirenal abscesses, gallbladder wall thickening, periportal edema, pleural effusion, and thickened interlobular septa [3]. Periportal edema, characterized by a zone of low attenuation surrounding the intrahepatic portal veins on contrast-enhanced CT scans, is a major extrarenal finding in acute pyelonephritis; notably, Compo et al. reported an incidence of 36% for this condition [2]. Although periportal edema also manifests in conditions such as congestive cardiac failure, acute viral hepatitis, blunt hepatic trauma, and lymphatic obstruction by primary liver tumors or periportal lymphoma, its prevalence in acute pyelonephritis necessitates specific attention in a clinical setting [4]. The pathophysiology of periportal edema in acute pyelonephritis remains unclear. The related discussions have revolved around altered sodium reabsorption, which has been attributed to the infectious process within the renal interstitium and the exacerbation of vascular permeability during severe systemic sepsis [3]. Notably, right-sided acute pyelonephritis cases more frequently exhibit periportal edema, which is likely due to the anatomical proximity of lymphatic connections that facilitate the asymmetrical spread of pathogens or inflammatory substances [5]. The presence of periportal edema in patients with acute pyelonephritis is associated with more severe clinical outcomes, including elevated CRP levels, the presence of leukocytes in urine, extended hospitalization, and substantial renal parenchymal involvement [6]. These findings underscore how identifying periportal edema in imaging studies is crucial for the accurate diagnosis and management of acute pyelonephritis.

## Data Availability

The data presented in this study are available on request from the corresponding author due to privacy.

## References

[1] El-Ghar M.A., Farg H., Sharaf D.E., El-Diasty T. (2021). CT and MRI in Urinary Tract Infections: A Spectrum of Different Imaging Findings. Medicina.

[2] Campos F.d.A., Rosas G.d.Q., Goldenberg D., Szarf G., D’Ippolito G. (2007). Acute pyelonephritis: Frequency of findings in patients submitted to computed tomography*. Radiol. Bras..

[3] Zissin R., Osadchy A., Gayer G., Kitay-Cohen Y. (2006). Extrarenal manifestations of severe acute pyelonephritis: CT findings in 21 cases. Emerg. Radiol..

[4] Karcaaltincaba M., Haliloglu M., Akpinar E., Akata D., Ozmen M., Ariyurek M., Akhan O. (2007). Multidetector CT and MRI findings in periportal space pathologies. Eur. J. Radiol..

[5] Shin J.S., Sung D.J., Park B.J., Kim M.J., Cho S.B., Han N.Y., Lee N.J. (2012). Gallbladder Wall Thickening and Periportal Tracking on CT in Patients with Acute Pyelonephritis. J. Korean Soc. Radiol..

[6] Vollmann R., Schaffler G.J., Spreizer C., Quehenberger F., Schoellnast H. (2011). Clinical significance of periportal tracking as an extrarenal manifestation of acute pyelonephritis. Abdom. Imaging.

